# Oral and dental health in Huntington‘s disease - an observational study

**DOI:** 10.1186/1471-2377-13-114

**Published:** 2013-09-03

**Authors:** Carsten Saft, Jürgen E Andrich, Thomas Müller, Julia Becker, Jochen Jackowski

**Affiliations:** 1 Department of Neurology, Huntington-Center NRW, St. Josef Hospital, Ruhr University, Gudrunstrasse 56, Bochum, 44791, Germany; 2 Department of Oral Surgery and Dental Emergency Care, Dental School, Faculty of Health, Witten/Herdecke University, Witten, Germany

## Abstract

**Background:**

Only a few case reports and case series dealing with oral and dental health care are available in literature until now. The aim of the present pilot study was to determine the status of dental health in comparison to matched controls and to heighten the neurologists’ and dentists’ awareness of the oral aspects of the disease.

**Methods:**

42 Huntington’s disease (HD) participants were scored according to the Unified Huntington’s Disease Rating Scale. The dental status was assessed by using the well established score for decayed, missing, and filled teeth (DMFT) and the dental plaque score (Silness-Loe plaque index).

**Results:**

Compared to controls HD participants showed significantly more decayed teeth and more plaques in both plaque indices. A higher motor impairment and a lower functional status of the patients lead to a worsening in dental status.

**Conclusion:**

Possible reasons for our findings are discussed. Apart from local oral complications general complications may also occur. Thus, as a consequence, we would encourage patients, caregivers, neurologists, and the dentists to ensure regular preventive dental examinations and dental treatments of individuals with Huntington’s disease even in the premanifest stage of this disease.

## Background

**Figure 1:**
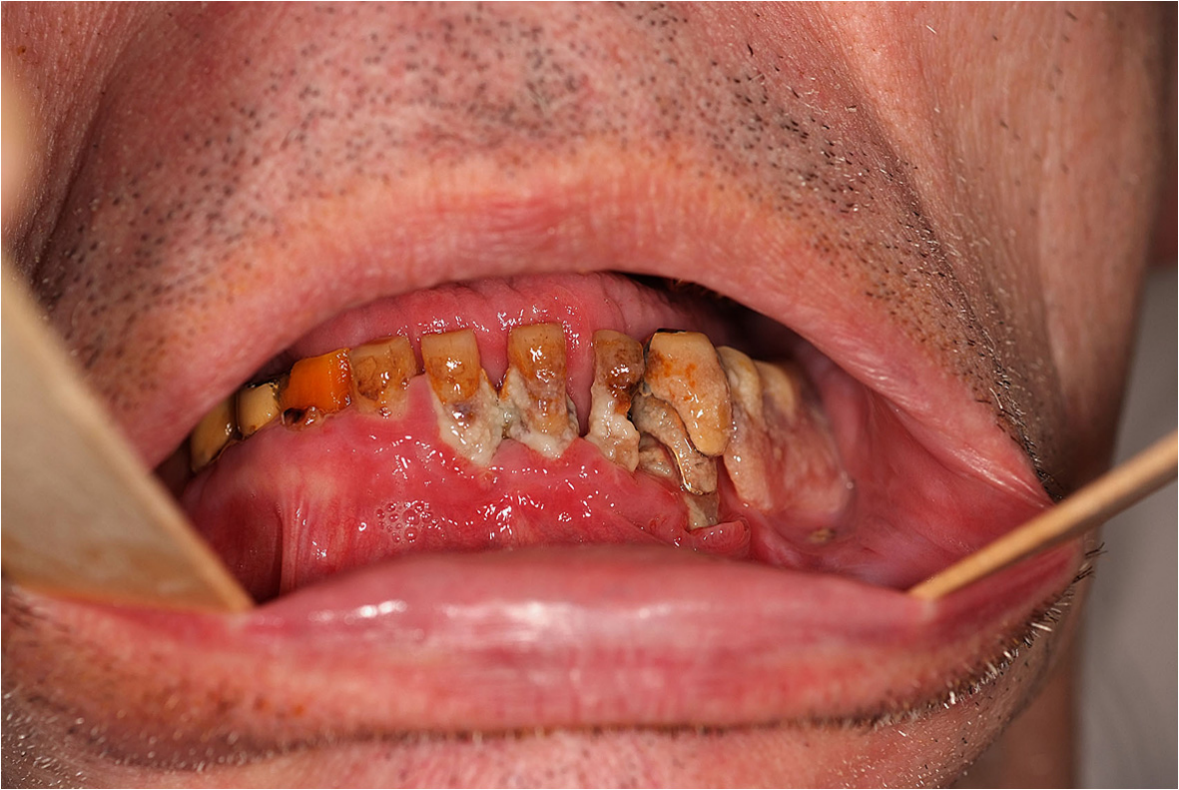
**Example of a disastrous dental status in the lower jaw of a 71 year old patient suffering from Huntington’s disease for five years not included in the study.** Extensive parodontitis, avital and carious teeth with plaques and osseous involvement are obvious. This patient did not complain of any dental pain and refused treatment.

Only a few data exist to indicate oral and dental health in patients with Huntington’s disease (HD). A search in PubMed/MEDLINE using the terms “Huntington dental”, “Huntington teeth” or “Huntington tooth” provided ten hits dealing with this subject [1-10]. Some of these cases report on different anesthesia techniques in order to enable dental treatment and/or the treatment of the single HD cases (e.g. by using implant-supported denture). To our clinical impression dental health care seems to be extremely insufficient in a large number of subjects suffering from HD (see Figure 1).

Problems caused by HD are accentuated by the dentist’s lack of neurological knowledge (i.e. pharmacological therapy, clinical fluctuations of drug therapy, and behavioural aspects) and the neurologist’s lack of awareness of many of the pathological changes in the oral cavity (i.e. oral and dental side effects and interactions of drugs used to treat HD, etiology of gingivitis, parodontitis, and caries).

Jackowski and colleagues stated in their case report that patients with extrapyramidal diseases, such as HD, are often not capable of maintaining independent and efficient oral hygiene due to restricted motor ability of the upper extremities and lack of coordination. The hermetic closure of the mouth and lips and the associated ability to keep liquid and toothpaste in the mouth might be impaired and, thus, an effective oral hygiene cannot be maintained. Moreover dyskinesia and hyperkinesia of the tongue and of the perioral musculature, combined with xerostomia and pooling of saliva, may have an influence. Finally, the different factors lead to a poor oral health status and loss of teeth. In the reported case an implant-supported complete denture led to a clear improvement in the patient's chewing function [3].

Deniz and colleagues mentioned cognitive decline and behavioural manifestations of the disease as well as medication side-effects as possible additional reasons contributing to a poor dental care status. In this case report, the patient was rehabilitated with a mandibular overdenture supported by two endosteal implants [2].

Based on our observations and the above mentioned case reports, the aim of this study was to evaluate the status of dental health in comparison to matched controls in HD.

## Methods

42 HD participants and matched healthy controls were included in the study. Controls were recruited from partners of the patients or via an announcement. Diagnosis of HD was confirmed by molecular genetic testing in 41 patients, three subjects were premanifest mutation carriers. One patient was diagnosed clinically and by a positive family history. Subjects with symptoms of a severe dementia (Mini-Mental State examination < 10) or other severe concomitant disorders, influencing their ability to declare consent, were excluded. The mean duration of the disease was 6.3 years. HD-symptoms were scored according to the Unified Huntington’s Disease Rating Scale (UHDRS), including the subscales for motor symptoms (MS), total functional capacity (TFC) and Independence scale (IS). Premanifest mutation carriers were defined by a positive gene test and absence of unequivocal motor symptoms according to the UHDRS diagnostic confidence level (DCL: < 4) [11]. The Mini-Mental State examination (MMSE) was performed for assessment of the cognitive status of the participants [12]. Recruitment of the subjects was carried out blinded to the dental status of the participants. Detailed data of subjects are given in Table 1.

**Table 1:** Clinical characteristics of HD participants and controls

	**HD**	**Controls**
**Age**	45.3±10.7	45.45±11.1
	(26–68)	(26–68)
**Sex (male/female)**	21/21	21/21
**Age of onset**^a)^	39.0±11.3	
**motor symptoms**	(20–63)	-
**Age of onset**	38.0±11.1	
**psychiatric symptoms**^b)^	(20–63)	-
**Duration of motor symptoms**^a)^	7.31±3.72	-
	(2–18)	
**Duration of psychiatric**	7.72±4.67	-
**symptoms**^b)^	(1–22)	
**UHDRS MS**	45.3±21.1	-
	(0–81)	
**UHDRS TFC**	7.5±3.2	-
	(2–13)	
**UHDRS IS**	70.0±16.5	-
	(30–100)	
**MMSE**	24.4±6.4	-
	(11–35)	

Values are given as mean and standard deviation, as well as the range in brackets. *Abbreviations:* UHDRS, unified Huntington’s disease rating scale; IS, independence scale; TFC, total functional capacity; MS, motor score; MMSE, mini-mental state examination. Missing data for the three premanifest participants^a)^; Missing data for 11 participants^b)^.

The two established World Health Organization-approved dental indices for decayed, missing, and filled teeth (DMFT; 1938) and the dental plaque score (PI) developed by Silness and Loe (1964) were selected to quantify dental disease [13-15].

DMFT was used to assess the number of teeth with caries, including the number of present teeth, missing teeth, sound teeth, treated teeth, untreated teeth, and the total number of carious teeth (DMF teeth: decayed, missing, and filled teeth). For the PI score teeth surfaces were given a score from 0 (no plaque) to 3 (abundance of soft matter within the gingival pocket and/or on the tooth and gingival margin). The index was obtained by calculating the mean for all investigated teeth and surfaces. PI-I was used for the mean of all teeth of the patient, PI-II for the mean of defined index-teeth. The teeth 17, 16, 11, 24, 26, 27, 37, 36, 31, 44, 46 and 47 were used for analysis according to the method described before [13]. If one of the index teeth was missing, the next adjacent tooth wase used for evaluations. Plaque indices were only possible to assess for 41 HD participants, because one patient refused further investigation.

Statistical analysis was performed using the commercial software program SPSS statistics. All measured parameters and clinical data were first analysed descriptively and are presented as mean ± SD. Normality of distribution of the data was tested using the Shapiro-Wilk-test. Independent t-test, respectively Mann–Whitney-U-test, was used to test for differences between the two groups. Bonferroni correction was used for multiple testing. Spearman-Rho correlation analysis was used for exploratory statistical correlation analysis. All participants gave written informed consent.

### Ethics

The local ethics committee of the Ruhr University Bochum approved this study.

## Results

HD participants showed significantly more decayed teeth than controls. Differences in the DMFT score for missed and filled teeth, however, failed to reach a significant level. HD participants also showed significantly more plaque in both plaque indices compared to controls. All results of DMFT score and the plaque indices are given in Table 2 in detail.

**Table 2:** Dental status of HD participants and controls assessed by the DMFT score (1938) for decayed, missing and filled teeth and Plaque indices score after Silness and Loe (1964)

	**HD**	**Controls**	**Significance**
**DMFT decayed**	2.98±3.10	0.71±1.09	**< 0.05**
	(0–10)	(0–4)	
**DMFT missing**	4.88±6.84	4.12±5.83	NS
	(0–28)	(0–28)	
**DMFT filled teeth**	10.29±5.43	12.02±5.02	NS
	(0–22)	(0–23)	
**DMFT sum score**	18.14±6.13	16.88±6.06	NS
	(6–28)	(3–28)	
**Plaque Indices I**^a)^	1.88±0.89	0.95±0.62	**< 0.05**
	(0.14-3.0)	(0–2.60)	
**Plaque Indices II**^a)^	1.92±0.89	1.15±0.64	**< 0.05**
	(0.14-3.0)	(0–2.50)	

^a)^Plaque indices were assessed for only 41 HD participants.

No differences between male and female HD participants were found. Explorative correlation analysis of DMFT and plaque indices (PI) results given in Table 2 with clinical symptoms showed no significant correlation with any of the clinical characteristics from Table 1, except for the UHDRS motor score and missing teeth (p 0.025, r .345), PI-I (p 0.009, r .404) and PI-II (p 0.006, r .426). The age of onset of motor symptoms showed a significant correlation with missing teeth (p 0.006, r .432), as did the psychiatric onset (p 0.020, r .416) and the TFC score correlated negative with the plaque scores (PI-I: p 0.001, r - .501; PI-II: p 0.001, r - .503).

## Discussion

To our best knowledge, this, present study is one of only a few reporting on dental health in HD. The results support our hypothesis that the dental health status is impaired in HD compared to controls. In particular, HD participants showed significant more decayed teeth and plaques as controls. A causal relationship between the dental status and HD is only partly supported by the correlation between UHDRS subscales and scores of the DMFT and PI scores. A lower functional status of the patients lead to a worsening in dental health (higher plaque scores). There was, however, no correlation between a higher plaque score or decayed teeth and the UHDRS motor score. Thus only an indirect interrelation between dental health and movement disorders could be found. We did not analyse the chorea subscore and parameters of dental health. The weak positive correlation with the age of onset might reflect an additional effect of age. To our surprise, there was no correlation between dental status and duration of the disease, which might be explained by an impaired oral health in the premanifest stage already.

An inefficient oral hygiene due to restricted motor ability of the upper extremities, a lack of coordination and/or an impairment of the hermetic closure of the mouth and lips due to more dys- and hyperkinesia of the tongue and of the perioral musculature might contribute to the impaired dental health status in HD [3]. We could not reveal any correlation between dental status and the mini-mental score and, therefore, we are not able to underline the thesis of cognitive decline as a reason for an impaired teeth status. This might also be due to the relative small number of patients investigated in this pilot-study. Because of the small number of participants we neither analyzed medication or smoking as possible influencing factors, nor did we analyze behavioural manifestations of the disease (e.g. using an apathy score) as reasons for the poor dental status, which is a clear limitation of our study. We also did not ask how frequently the patients cleaned their teeth compared to controls. All factors, however, might contribute, especially apathy may be a reason for a lack of dental hygiene even in premanifest stages of the disease [16,17]. Moreover, an impaired awareness of a poor dental status might be an explanation, since an impaired awareness of motor, functional and cognitive deficits is known in HD [18,19]. As an additional possible reason an altered sensory processing and lack of pain recognition might contribute [20,21]. Salivation can be altered due to medication effects but also due to altered autonomic function and hormonal influences [22-25]. In more advanced stages of the disease also dysphagia, vomiting and regurgitation influences the dental status [26]. Thus, influences from the disease itself as well as secondary mechanisms like malnutrition, medication and general disability may contribute.

We also found a lower score for filled teeth, that is for treated teeth by a dentist and a higher level of missing teeth in the HD group. Both findings, however, failed to reach statistical significance. The lower scores might be explained by a higher rate of tooth extraction or due to trauma and the consequence of falls, which is supported by the correlation of missing teeth with the UHDRS motor score. A poorer dental status may lead earlier to a tooth extraction as the only causal therapy in HD patients.

It is discussed whether the dental care status predicts circulatory mortality and other causes of death. As possible pathways, the effects of masticatory dysfunction on dietary behaviour, nutrition and systemic diseases and inflammatory effects on the circulatory system are discussed [27-29]. Oral disease was found to be associated with excess cardiovascular disease risk, with possibly common pathogenetic mechanisms between poor oral health and cardiovascular disease [30].

In some cases "a bad tooth" was discussed as the cause of premature death (e.g. in the case of Theodore Roosevelt (1858–1919) the twenty-sixth President of the United States who anecdotally died from oral sepsis) [31,32]. Thus, beside local complications it cannot be excluded that the impaired dental status in HD leads to an earlier death directly or by triggering cardiovascular diseases [33,34].

## Conclusions

Preventive dentistry is the most important aspect of dental treatment for the person with Huntington’s disease, as with all patients. Although the goals of preventive dentistry include self-sufficiency, the spouse or caregiver must be educated to supervise and evaluate hygiene procedures daily at home. As HD progresses, patients become increasingly incapable of completing even the simplest forms of oral care. The primary goals of dental treatment planning should be maintenance of natural dentition, early intervention, regular preventive treatment and preservation of self-care. HD subjects in any stage of the disease should see a dentist at least every 4 months whether they have natural teeth, complete dentures, or a mixture of teeth and dentures. Pathological conditions may be diagnosed early and treated in simple stages. When clinical findings suggest Huntington’s disease, the patient should be referred for oral and dental evaluation. Oral health parameters, including salivary flow gingival health (bleeding, plaque, calculus), periodontal health (pocket depth, recession, loss of attachment), and dental condition (number of teeth, decay, restored), must be measured at onset (baseline) and course of the disease. Some authors also recommend to visit the same dentist over time, if possible [4].

## Competing interests

Carsten Saft received honorarium from Temmler Pharma GmbH & Co..KG for scientific talks, compensation in the context of the Registry-Study of the Euro-HD-Network, in the context of the ACR16-Study (Neurosearch), the AFQ-Study (Novartis), the Selisistat-Studies (Siena Biotech) and received research support for a research project with Teva Pharma GmbH and the 'Cure Huntington's Disease Initiative (CHDI Foundation, Inc.). Julia Becker, Thomas Müller, Jürgen Andrich and Jochen Jackowski has nothing to declare. The authors declare that they have no competing interests.

## Authors’ contributions

Study concept and design: JEA, TM and JJ. Acquisition of data: CS, JB and JEA. Analysis and interpretation of data: JB, CS and JEA. Statistical analysis: JB and CS. Drafting of the manuscript: CS. Critical revision of the manuscript for important intellectual content: JEA, TM and JJ. All authors read and approved the final manuscript.

## Pre-publication history

The pre-publication history for this paper can be accessed here:

http://www.biomedcentral.com/1471-2377/13/114/prepub
